# Near‐harvest application of methyl jasmonate affected phenolic content and antioxidant properties in “Thompson Seedless” grape

**DOI:** 10.1002/fsn3.2697

**Published:** 2022-01-05

**Authors:** Ehsan Ranjbaran, Mansour Gholami, Martin Jensen

**Affiliations:** ^1^ Department of Horticultural Science, Faculty of Agriculture Bu‐Ali Sina University Hamedan Iran; ^2^ Department of Food Science Aarhus University Aarhus N Denmark

**Keywords:** elicitor, free radicals, polyphenols, secondary metabolites, *Vitis vinifera*

## Abstract

The influence of methyl jasmonate (MJ) preharvest treatment was investigated on some polyphenols and antioxidant systems in the “Thompson Seedless” table grape. The clusters were sprayed in the vineyard 2 days before harvest with 0, 1, 5, and 10 mM MJ. After picking, berries were stored for 6 days at 15°C, simulating marketing conditions. Total phenols and flavonoids were affected by MJ treatment, especially at 10 mM concentration, whereas total tannins were found to be unchanged. Antioxidant activity of the treated skin was noticeably higher compared with the control, together with PAL and POD activity. Although MJ had little effect on catechin and epicatechin, the levels of quercetin and rutin were noticeable. In addition, 5 and 10 mM MJ exerted a pronounced effect on transresveratrol content. These data showed that a single preharvest application close to the harvest time could be an effective treatment to promote the antioxidant properties of the grape.

## Graphical abstract

Total phenol, total flavonoid, and antioxidant activity were enhanced by methyl jasmonate treatment. Contrary to flavanols, methyl jasmonate had a pronounced effect on flavonol and resveratrol content in treated grapes. A single treatment close to the harvest time was a cost‐effective strategy to improve enzymatic and nonenzymatic antioxidant properties in grapes.

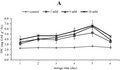

## 1 INTRODUCTION

Grapes, one of the most widely consumed fruits worldwide, are rich in phenolic compounds. These compounds in grapes are involved in qualitative and organoleptic characteristics and storability (Zhang et al., 2012). Grapes also consist of diverse kinds of phenolics contributing to improved human health (Vauzour et al., 2010; Vislocky & Fernandez, 2010; Xia et al., 2010). In view of all the aforementioned reasons, augmentation of these compounds is widely taken into consideration. In this regard, elicitation has been shown to be a strategy to stimulate the synthesis of these bioactives in fruits (Ruiz‐García & Gómez‐Plaza, 2013). Naturally occurring compounds, meanwhile, have been extensively noticed to enhance phenolic content in table grapes, such as abscisic acid (Koyama et al., 2018), salicylic acid (Champa et al., 2015; Ranjbaran et al., 2011), brassinolide (Xi et al., 2013), and methyl jasmonate (MJ; Portu et al., 2017).

Methyl jasmonate, a signal molecule applied as a chemical elicitor, induces plant defense mechanisms, leading to the synthesis of secondary metabolites (Beckers & Spoel, 2006). MJ has also been shown to affect antioxidant and defense enzymes involved in phenolic metabolism, including phenylalanine ammonia lyase (PAL), polyphenol oxidase (PPO), and peroxidase (POD) (Belhadj et al., 2006). Hence, the effect of MJ application on phenolic content and antioxidant activity to different fruits, such as raspberry (Flores & del Castillo, 2015), black currant (Flores & Ruiz Del Castillo, 2016), sweet cherry (Castillo et al., 2014; Saracoglu et al., 2017), kiwifruit (Öztürk & Yücedağ, 2021), medlar (Ozturk et al., 2019), plum (Karaman et al., 2013), apple (Ozturk et al., 2013), and grape wine (Portu et al., 2015) has been studied. In this context, MJ has most often been applied several times at veraison in grapes, which is considerably expensive (Garde‐Cerdan et al., 2016; Portu et al., 2016; Ruiz‐García et al., 2013). It has been shown that the content of jasmonates in grape skin decreases along with ripening (Kondo & Fukuda, 2001). Likewise, a significant decline in phenolic content of grape skin during ripening has been reported (Obreque‐Slier et al., 2010), which might be associated with the mitigation of jasmonates in this process. For this reason, the application of jasmonates as a supplement near harvest time could be effective to improve berry phenolics.

To our knowledge, little information is available on a single MJ treatment as a supplement near harvest time, evaluating the residual effects on phenolic metabolism in grapes, especially table grapes, after harvest. Therefore, this work aimed to assess the impact of MJ in three different concentrations (1, 5, and 10 mM) with control applied 2 days before harvest on polyphenols and antioxidant capacity on the “Thompson Seedless” table grapes.

## 2 MATERIAL AND METHODS

### 2.1 Chemicals

Methyl jasmonate, 2,2'‐azino‐bis(3‐ethylbenzothiazoline‐6‐sulphonic acid) (ABTS), HCl 37%, 2,4,6‐Tris (2‐pyridyl)‐s‐triazine (TPTZ), DPPH radical (diphenyl‐1‐picrylhydrazyl), (+)‐catechin, (−)‐epicatechin, quercetin 3‐*O*‐glucoside, and transresveratrol were obtained from Sigma‐Aldrich (US). *L*‐phenylalanine, 2‐methoxyphenol (Guaiacol), pyrocatechol, iron (III) chloride hexahydrate (FeCl_3_ (6H_2_O)), potassium persulfate (K₂S₂O₈), iron (II) sulfate heptahydrate (FeSO_4_ (7H_2_O)), sodium nitrite, Tween‐80, hydrogen peroxide 30%, Folin–Ciocalteu reagent, and polyvinylpolypyrrolidone (PVP) were purchased from Merck. Acetonitrile and rutin trihydrate were purchased from CHEMSOLUTE and Fluka, respectively. Trifluoroacetic acid (TFA) and methanol (all of HPLC grade) were obtained from VWR CHEMICALS. Milli‐Q water was acquired by SG water apparatus.

### 2.2 Field treatments and storage

The experiment was carried out at Malayer Grape Research Station located in Hamedan, Iran (34º15′49.2′′N, 48º48′09.4′′E), using “Thompson Seedless” grapes (*Vitis vinifera* L.). MJ solution in different concentrations (0, 1, 5, and 10 mM) was prepared in water and Tween‐80. All treatments were applied 2 days before harvest by spraying on the clusters to run‐off. To determine berry maturity, the total soluble solids (TSS) of the grape juice were controlled. Commercially matured clusters were picked and transferred immediately to the lab.

After one night precooling, intact berries with homogeneous size were selected randomly from the whole part of each bunch. About 35 clusters were allocated to each treatment. Each replicate was kept in perforated plastic clamshell containers at 15°C and 80% RH for 6 days in darkness. Twenty healthy berries were selected out of each container every day, peeled, and the skin was ground to a powder with liquid nitrogen before keeping at −80°C.

### 2.3 Determination of phenolic content

Extraction of phenolic compounds was performed by the method of Li et al. (2015). Briefly, the grape powder was macerated in the extraction solvent including methanol/acetone/water (3.5:3.5:3, v/v/v) with 1% (v/v) acetic acid glacial, and centrifuged after shaking for 30 min in the dark at room temperature (RT). The supernatant was applied for phenolic and antioxidant measurements.

Total phenol content (TPC) of the extract was determined as described by Slinkard and Singleton (1977) and expressed as mg gallic acid equivalent (GAE) per gram fresh weight (fw). Shortly, an aliquot of the extract and Folin's reagent were mixed with sodium carbonate, shaken for 90 min, and then the absorbance was read at 765 nm by a spectrophotometer (Varian Cary 100 UV‐Vis, US).

Total flavonoid content (TFC) of the extract was measured as developed by Yoo et al. (2008) and expressed as mg rutin equivalent (RE) per g fw. In short, an aliquot of the extract was diluted with distilled water and mixed with sodium nitrite 5%, aluminum chloride 10%, and sodium hydroxide. The absorbance of the final solution was recorded at 510 nm.

For total tannin content (TTC), the Folin–Denis method was followed (Taira, 1995) and expressed as mg tannic acid equivalent (TAE) per g fw. Briefly, diluted Folin's reagent 1 N was mixed with saturated sodium carbonate, shaken for 60 min, and read spectrophotometerically at 725 nm.

### 2.4 Determination of antioxidant activity

The antioxidant activity (AA) of the extracts was determined via three different methods. DPPH radical scavenging potential was measured as proposed by Bertelli et al. (2014). An aliquot of the skin extract and/or water (as control) was mixed with DPPH radical solution, vortex‐mixed well, and kept for 30 min at RT in the dark. The absorbance was read at 515 nm and the result was expressed as a percentage of inhibitory capacity of DPPH radical.

The ABTS assay was carried out following the method developed by Szymanowska et al. (2015). For making the ABTS radical solution, ABTS 7 mM was mixed with potassium persulfate 2.45 mM (1:1) and incubated at RT overnight. The working mixture was diluted to make an absorbance of 0.911 at 734 nm. The extract was then mixed with ABTS radical solution. Distilled water was considered as a control. The ABTS radical scavenging ability of extract was expressed as a percentage of inhibitory capacity of ABTS radical.

FRAP assay was performed as followed by Benzie and Strain (1996). In brief, the working solution was prepared by mixing acetate buffer (300 mM, pH 3.6), TPTZ 10 mM solution with HCl 40 mM, and FeCl_3_‐6H_2_O 20 mM (10:1:1 v/v/v). Then, the extract was mixed with the working solution and incubated at 37°C in a bain‐marie for 10 min. The absorbance was recorded at 593 nm and the result was expressed as the ability to reduce 1 mM equivalent Fe(+2) to Fe(+3) per g fw.

### 2.5 Determination of enzyme activity

For enzyme extraction, 400 µg of the frozen sample was suspended in extraction solution including phosphate buffer (0.05 M, pH 6.8), triton X‐100 (0.05% v/v), 2‐mercaptoethanol (5 mM), and PVP (0.1% w/v) (Galli et al., 2009). This mixture was centrifuged and the clear supernatant was collected as enzyme extract. The whole process was carried out at 4°C.

The PAL activity was evaluated according to Zhu et al. (2016). Two hundred microliters of the enzyme extract was diluted by 0.2 M borate buffer. Then, 0.02 M *L*‐PA was added, and this mixture was left to incubate at 37°C. After 30 min, 6 M HCl was added. The absorbance of the samples was recorded at 290 nm.

The activity of PPO and POD was measured as described by Christopoulos and Tsantili (2015), but with slight modifications. For the PPO activity assay, the crude extract was diluted with 0.05 M phosphate buffer (pH 6.2). Before 2‐min incubation at 25°C, catechol solution (0.05 M) was added to this mixture. Finally, the changes in absorbance were followed at 420 nm.

The POD activity was analyzed by guaiacol oxidation. The reaction solution containing the extract, 0.025 M guaiacol (in 0.1 mM phosphate buffer, pH 6) and H_2_O_2_ 0.020 M, was incubated for 2 min at 25°C while monitoring the changes in absorbance spectrophotometerically at 470 nm.

The activity of the enzyme was expressed as an enzyme unit (the enzyme content leading to an increase of 0.001 absorbances at the relative wavelength per min under the circumstances described) per g fw.

### 2.6 HPLC analysis

Extraction for phenol compositional analysis was performed as follows: 300 µg of the freeze‐dried sample was homogenized by acidified methanolic solvent (MeOH 85% in water, HCl 0.1%). This mixture was sonicated (5°C, 20 min), shaken (1,000 rpm, 10°C, 20 min), macerated (overnight, 4°C), and then centrifuged (10,000 × *g*, 10 min, 4°C). The supernatant was filtered by 0.45 µm Q‐Max^®^ membrane filter and kept at −18^◦^C for HPLC analysis.

Phenolic compounds were analyzed using an HPLC (Ultimate 3000, Dionex), equipped with a Corona^®^ CAD^®^ (Charged Aerosol Detector), an automatic sampler, and a four‐channel pump. The injection volume was 10 µl. Separation of compounds was accomplished by a 250 × 4.6 mm, 5 µm, RP‐18 column (Hypersil GOLD^TM^, Thermo Scientific, US) adjusted at 30^◦^C. TFA 0.1% in water (eluent A) and acetonitrile (eluent B) were used as mobile phases with a flow rate of 1 ml/min. The gradient began with 5% eluent A, reaching 30% at 20 min, 40% at 25 min, 100% at 35 min, and 5% at 37–45 min. The compounds were identified according to the retention time and their spectra. The amount of phenolic compounds was determined by using the corresponding standard curve.

### 2.7 Statistical analysis

Effects of MJ and storage time were determined by ANOVA. Significant difference among treatments was assessed according to Duncan's multiple tests (*p* ≤ .05). All analyses were carried out using SAS version 9.4 (SAS Institute Inc., Cary, NC, USA). All measurements were performed in triplicate.

## 3 RESULTS AND DISCUSSION

### 3.1 Phytochemical analysis

**FIGURE 1:**
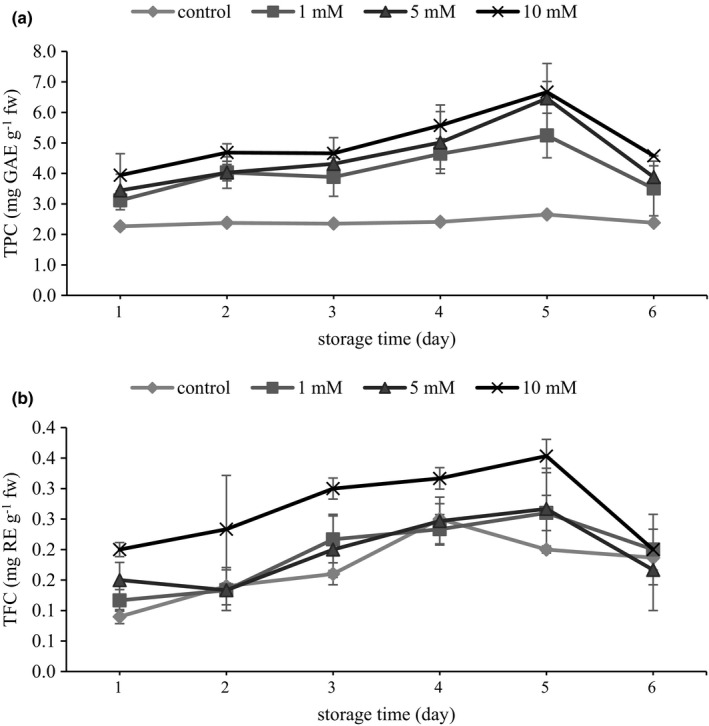
Effect of preharvest methyl jasmonate treatment on TPC (a) and TFC (b) of grape berry skin during storage. Values are the mean ± standard error of three replicates

As seen for total phenol in Figure 1. A, all MJ‐treated skins had predominantly high TPC with respect to the control. Contrary to the control which was unchanged over time (ca. 2.4 mg GAE g^−1^ fw), TPC in all of the treated samples followed an increasing pattern till day 5, when 5 and 10 mM MJ presented the highest TPC (2.5‐fold that of control) (Figure 1a). As proposed before, such enhancement of TPC could contribute to the activity of biosynthetic enzymes, especially PAL, in response to exogenously applied MJ (Wang et al., 2009). In another study published by Flores et al. (2015), in accordance with our result, postharvest application of a racemic mixture of MJ was established to boost TPC and antioxidant activity in treated grapes.

In the case of flavonoids, on the first day of sampling, MJ skins, in a concentration‐dependent manner, had higher TFC relative to the control, where 10 mM MJ (0.2 mg RE g^−1^ fw) contained the highest flavonoid (Figure 1b). Despite the MJ treatment, TFC increased along with storage in the treated and no treated skins; the difference is that TFC in the control skin reached the maximum 1 day earlier (day 4) than those of treated ones (day 5). As can be seen (Figure 1b), 10 mM MJ (0.35 mg RE g^−1^ fw) appreciably showed higher TFC with respect to the other concentrations and control (1.75‐fold higher than control). Osturk et al. (2015) investigated the effect of MJ preharvest application on TFC in Japanese plum (Ozturk et al., 2015). Likewise, flavonoid accumulation after preharvest MJ treatment has already been reported in grapes (Ruiz‐Garcia et al., 2012), blackberries (Wang et al., 2008), and blueberries (Percival & MacKenzie, 2007). Increased phenolics during the short storage, which has previously been noticed in grapes (Maurer et al., 2017), could be a resultant organic acid decomposition providing carbons needed for phenolic formation (Kalt et al., 1999). In the present study, however, all samples subsequently experienced a reduction at the end of storage, which was slower for those of control than treated skins. The reversal of trend in TPC and TFC during storage in both treated and control berries after 5 days may be owing to the degradation and oxidation process backed by PPO and POD (Huang et al., 2015).

In terms of tannin, no significant differences were found when treated and control skin was evaluated during the storage time (data not shown). One of the important attributes of ripe table grapes is tannin‐related astringency. Condensed tannins are commonly comprised of catechin and epicatechin monomers, and the complex structure resulted from these compounds and proanthocyanidins (Zucker, 1983). Due to their effect on the tannin content in wines, comprehensive studies have been done on wine grapes (Gawel et al., 2001). In contrast, astringency is an undesirable feature in table grapes (Dokoozlian, 2000). Therefore, our result suggested that taste of the berries might not be affected by MJ after harvest. Moreover, following a clone‐dependent manner, it has been proposed that other phenolics could be favored at the expense of tannins (Portu et al., 2015). In this regard, our results showed that preharvest application of MJ increased flavonoid content, while no significant change was detected for tannin content.

### 3.2 Antioxidant analysis

**FIGURE 2:**
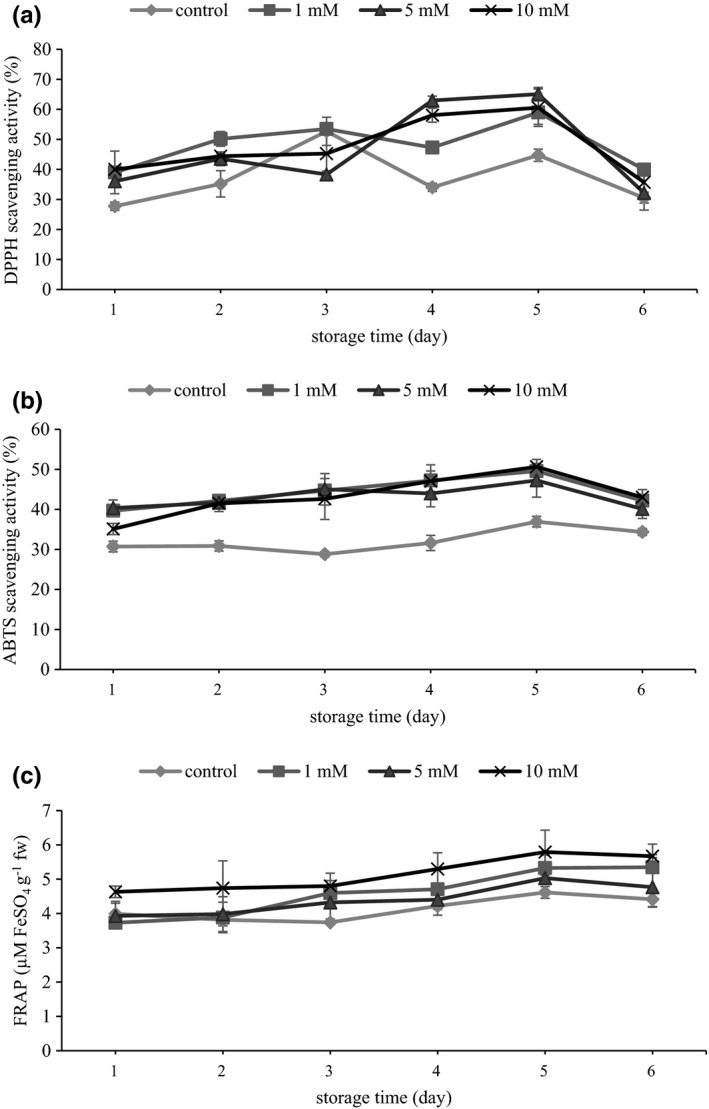
Effect of preharvest methyl jasmonate treatments on AA according to the DPPH (a), ABTS (b), and FRAP (c) of grape skin during storage. Values are the mean ± standard error of three replicates

As presented in Figure 2. A, DPPH scavenging activity of the skins increased over time for all samples. Nevertheless, control samples experienced the maximum value earlier (day 3) relative to the treated samples (day 5). On the other hand, except day 3, control samples had less AA than treated ones, especially on day 5, when 5 mM MJ obtained the highest DPPH scavenging activity (1.5‐fold higher than control) (Figure 2a).

As regards ABTS assay, AA of all samples showed an increasing trend throughout storage time, although treated berries showed noticeably higher AA compared with the control from the first day of storage until the end (Figure 2b). This increasing trend is in accordance with TPC.

As regards FRAP assay, 10 mM MJ depicted higher AA than the other treatments and control on the first day of measurement. With the progress of time, the AA of 1 and 5 mM was distinct from that of control. Notwithstanding the treatment, similar to the ABTS assay, AA of all samples increased during storage (Figure 2c).

These findings support the fact that MJ‐treated grape extract limits the effectiveness of free radicals in oxidative reactions (Flores et al., 2013). Similar to the present results, it has been previously shown that pre‐ and postharvest application of MJ on apple (Ozturk et al., 2015), Chinese bayberry (Wang et al., 2009), and table grape (Jiang et al., 2015) leads to an increment of AA in fruits throughout storage. Flores and Ruiz Del Castillo (2016), after using different concentrations of MJ before harvest, found that higher scavenging activity of MJ‐treated blackcurrants was due most likely to higher levels of flavonoids. This finding could be explained by the presence of the hydroxyl groups in flavonoids enabling them to neutralize free radical activity (De León et al., 2013; Martínez‐Flórez et al., 2002). For this reason, enhanced AA of MJ‐treated grapes might contribute to higher flavonoid content observed in this work.

In addition, it has already been indicated that the antioxidant potential of horticultural products is associated with phenolic content (Heredia & Cisneros‐Zevallos, 2009; Saracoglu et al., 2017). Advocating for this idea, AA in our study followed the same trend as phenolic content did during storage. The amplifying effect of MJ on enzymes involved in the phenylpropanoid pathway has been well documented (Kim et al., 2006; Repka et al., 2004). However, Saracoglu et al. (2017) demonstrated that preharvest application of 10 mM MJ at 3 weeks before harvest time resulted in a reduction in phenolic content and AA of sweet cherry fruits, and indicated that the effect of MJ on polyphenols might be changed with the time of treatment.

With high levels of AA, these findings in our study suggest enhanced remedial potential of MJ‐treated grapes, when compared with the control.

### 3.3 Enzyme analysis

Changes in PAL activity were significant over storage time (*p* < .0001). The effect of MJ on PAL activity can be detected from day 3, being higher in 1 mM MJ than others. Regardless of treatment, in all of the samples, PAL activity increased throughout storage up to day 5 and then decreased. Berries treated with 5 mM MJ showed a slight decrease at the end and had higher PAL activity in comparison with other treatments and control. However, the effect of MJ was not significant on PAL activity (*p* = 0.6281) (Table 1).

**TABLE 1:** Changes in PAL, PPO, and POD activity (U g^−1^ fw.min) in the skin treated with preharvest methyl jasmonate during storage

Storage time (day)	Control	1 mM	5 mM	10 mM
PAL				
1	130.00 ± 18.93 b‐e	112.50 ± 7.22 de	116.25 ± 2.17 de	137.50 ± 15.88 a‐e
2	129.69 ± 4.51 b‐e	140.25 ± 11.69 a‐e	109.38 ± 1.80 e	118.75 ± 10.83 c‐e
3	131.25 ± 3.61 b‐e	156.25 ± 18.04 a‐e	131.25 ± 10.83 b‐e	116.25 ± 15.35 de
4	139.06 ± 6.31 a‐e	162.50 ± 14.43 a‐e	150.00 ± 7.22 a‐e	156.25 ± 8.86 a‐e
5	175.00 ± 50.52 a‐d	193.75 ± 3.61 a	191.67 ± 49.12 ab	187.50 ± 18.04 ab
6	120.83 ± 12.28 c‐e	120.83 ± 11.02 c‐e	179.17 ± 20.48 a‐c	105.00 ± 5.77 e
PPO				
1	15.63 ± 1.80 e‐h	18.42 ± 4.04 c‐h	18.75 ± 3.61 c‐h	18.33 ± 1.86 c‐h
2	13.58 ± 1.30 gh	16.67 ± 2.60 e‐h	15.42 ± 1.82 e‐h	18.08 ± 1.20 c‐h
3	13.63 ± 0.62 gh	19.75 ± 0.58 c‐g	14.58 ± 2.09 f‐h	19.67 ± 1.45 c‐g
4	17.38 ± 1.34 d‐h	22.38 ± 1.52 b‐e	23.75 ± 1.44 a‐d	20.83 ± 1.45 c‐f
5	24.69 ± 1.62 a‐c	18.75 ± 3.61 c‐h	29.69 ± 2.71 a	28.50 ± 2.08 ab
6	12.50 ± 1.15 hr	12.83 ± 2.17 gh	17.69 ± 0.61 d‐h	16.96 ± 1.67 d‐h
POD				
1	12.50 ± 1.30 jk	26.25 ± 1.44 e‐h	26.92 ± 1.62 e‐h	27.75 ± 1.59 e‐h
2	16.25 ± 1.73 i‐k	32.08 ± 1.74 c‐g	31.88 ± 3.25 c‐g	26.04 ± 1.99 e‐h
3	23.44 ± 0.90 g‐i	33.33 ± 1.82 c‐f	37.33 ± 1.74 b‐d	35.42 ± 1.50 b‐e
4	25.00 ± 3.61 f‐i	33.28 ± 3.70 c‐f	37.50 ± 4.02 b‐d	40.63 ± 1.08 a‐c
5	30.50 ± 0.29 d‐g	43.75 ± 7.22 ab	49.38 ± 1.80 a	47.50 ± 1.44 a
6	9.38 ± 1.80 k	26.58 ± 3.94 e‐h	20.00 ± 5.73 hr‐j	19.08 ± 1.74 hr‐j

Note

Values are the mean ± standard error of three replicates. Different lowercase letters indicate significant difference at *p* < .05 according to Duncan test.

In spite of MJ treatment, the PPO activity in all samples, treated and untreated ones, with an initial decrease started to increase over time and, finally, at the end of storage, declined. Until day 4, grapes treated with 10 mM MJ represented a higher PPO activity relative to other treatments and control. On days 5 and 6, berries treated with 1 and 5 mM MJ had lower PPO activity compared with 10 mM MJ and especially with control. Although enzyme activity declined at the end of storage, 10 mM MJ and control still were higher than other treatments (Table 1).

Surprisingly, MJ treatment led to a noticeably higher POD activity with respect to control; however, all samples presented an increasing trend during storage (Table 1).

PAL has already been well known to be an initial enzyme in the biosynthesis of phenols (Pina & Errea, 2008). According to the literature, PAL activity can be stimulated upon different stress conditions (Dixon & Paiva, 1995). Besides grapes, increased activity of PAL after following MJ treatment has already been reported in guava, blackberry, and radish sprout (González‐Aguilar et al., 2004; Kim et al., 2006; S. Y. Wang et al., 2008), all of which were coincident with our results. A similar result has been observed in an in vitro study where MJ induced PAL and other enzymes involved in the biosynthesis pathway (Belhadj et al., 2008). Flores and del Castillo (2014) observed an increase in flavonoid content of MJ‐treated raspberries; they attributed this induction to the effect of MJ on PAL activity. Therefore, high levels of TFC of treated berries in the present study could be elucidated by the elevated activity of PAL. In contrast, JA treatment hindered the expression of PAL and 4Cl in the biosynthesis pathway (Jacobo‐Velázquez et al., 2015).

It seems that most of the studies carried out on the effect of MJ on PPO and POD have been about browning. It has been shown that such defense enzymes catalyze hydroxylation and oxidation of phenolic compounds, resulting in tissue browning (Christopoulos & Tsantili, 2015; Teoh et al., 2016).

An increment in enzyme activity during storage might be due to a de novo increase in the activity of enzyme precursors. It also can be attributed to the regeneration of enzymes over time (Christopoulos & Tsantili, 2015). Reduction in such enzymes observed at the end of storage brings about ROS accumulation which cause damage to DNA and RNA, and also peroxidation of membrane followed by early senescence (Hodges et al., 2004).

Similar to the result presented here, it has been demonstrated that JA and MJ activate the stress response paths and consequently induce PPO and POD activity (Boughton et al., 2006).

### 3.4 HPLC analysis

For a better understanding of the phenolic composition and their metabolism, a detailed study was carried out through HPLC. Results of phenolic analysis are presented in Table 2.

**TABLE 2:** Changes in epicatechin, rutin, quercetin, and resveratrol content (mg/g fw) in the skin treated with preharvest methyl jasmonate during storage

Storage time (day)	Control	1 mM	5 mM	10 mM
Epicatechin				
1	0.417 ± 0.00 b‐f	0.362 ± 0.02 f	0.448 ± 0.02 a‐d	0.379 ± 0.00 ef
2	0.447 ± 0.03 a‐d	0.419 ± 0.02 a‐f	0.476 ± 0.01 a‐c	0.396 ± 0.02 d‐f
3	0.412 ± 0.02 c‐f	0.444 ± 0.03 a‐e	0.465 ± 0.04 a‐c	0.409 ± 0.02 c‐f
4	0.464 ± 0.01 a‐d	0.478 ± 0.05 a‐c	0.464 ± 0.01 a‐d	0.428 ± 0.01 a‐f
5	0.487 ± 0.00 a	0.485 ± 0.02 ab	0.487 ± 0.00 a	0.446 ± 0.02 a‐d
6	0.477 ± 0.01 a‐c	0.453 ± 0.01 a‐d	0.484 ± 0.00 ab	0.455 ± 0.02 a‐d
Rutin				
1	0.444 ± 0.07 abc	0.478 ± 0.04 abc	0.491 ± 0.01 abc	0.475 ± 0.06 abc
2	0.404 ± 0.06 bc	0.511 ± 0.04 abc	0.455 ± 0.00 abc	0.490 ± 0.08 abc
3	0.392 ± 0.01 bc	0.508 ± 0.06 abc	0.456 ± 0.02 abc	0.555 ± 0.01 ab
4	0.380 ± 0.10 c	0.559 ± 0.06 ab	0.504 ± 0.08 abc	0.452 ± 0.04 abc
5	0.367 ± 0.04 c	0.491 ± 0.04 abc	0.582 ± 0.01 a	0.477 ± 0.02 abc
6	0.380 ± 0.01 c	0.446 ± 0.07 abc	0.513 ± 0.04 abc	0.493 ± 0.03 abc
Quercetin				
1	3.068 ± 0.43 d	3.878 ± 0.40 a‐d	3.362 ± 0.04 cd	4.275 ± 0.44 a‐d
2	3.384 ± 0.45 cd	3.902 ± 0.29 a‐d	3.908 ± 0.22 a‐d	4.592 ± 0.54 a‐c
3	3.016 ± 0.26 d	3.880 ± 0.46 a‐d	3.747 ± 0.30 a‐d	4.845 ± 0.85 ab
4	3.266 ± 0.27 d	4.042 ± 0.02 a‐d	3.799 ± 0.08 a‐d	5.064 ± 0.56 a
5	4.139 ± 0.32 a‐d	4.657 ± 0.56 a‐c	4.034 ± 0.00 a‐d	4.242 ± 0.34 a‐d
6	3.528 ± 0.41 b‐d	3.513 ± 0.12 cd	3.870 ± 0.20 a‐d	3.804 ± 0.30 a‐d
Resveratrol				
1	nd	0.244 ± 0.05 ef	0.188 ± 0.02 ef	0.795 ± 0.10 cd
2	nd	0.144 ± 0.02 f	0.259 ± 0.07 ef	1.003 ± 0.02 bc
3	nd	nd	0.240 ± 0.07 ef	0.966 ± 0.21 bc
4	nd	nd	0.408 ± 0.03 e	1.174 ± 0.08 ab
5	nd	nd	0.704 ± 0.14 d	1.335 ± 0.21 a
6	nd	nd	0.664 ± 0.10 d	1.376 ± 0.08 a

Note

Values are the mean ± standard error of three replicates. Different lowercase letters indicate significant difference at *p* < .05 according to Duncan test.

According to ANOVA, catechin content was not affected significantly upon MJ treatment during storage (data not presented), which is inconsistent with the result of Portu et al. (2017) who found no difference between control and MJ‐treated grapes in the catechin and epicatechin content even when applying a precursor, phenylalanine. Furthermore, no response of such monomeric procyanidins to MJ treatment was detected in the “Fuji” apple (Rudell et al., 2002). As also with epicatechin, in the present study, the absence of difference between treatments and control was denoted. What is more, attenuation of the epicatechin was brought about by the highest dose of MJ (10 mM). The negative effect of MJ, as previously reported by Rudell et al. (2002), can be elucidated by MJ‐induced PPO activity (Masia et al., 1998). Concomitant with this result, Portu et al. (2015) demonstrated that foliar application of MJ had no effect on flavanols in grape berries and the wine made from them. In this vein, as mentioned earlier, flavonol biosynthesis enzymes could be activated instead of those for flavanol synthesis, which is clone dependent (Ruiz‐García et al., 2013). It should be noted that this explanation is in accordance with the result of tannin content.

Table 2 depicts the analysis of grapes from different MJ treatments and control on their quercetin content. As seen, compared with the control, all MJ grapes had higher quercetin content throughout storage until day 4, especially 10 mM MJ, being 1.5‐fold higher than control at day 4. Contrary to the other concentrations, 1 mM MJ‐treated skin had still higher quercetin content at day 5. At the end of the storage, however, no significant difference was detected between treatments and control. Of note, the effect of MJ on rutin content was somehow different, so that all MJ treatments had higher rutin values during storage relative to the control. In contrast to the control remaining low and constant, MJ grapes reached a peak on different days. As can be seen in Table 2, the residual effect of MJ on rutin was kept until the end of storage. In general, higher flavonol content was obtained from grapes that had been exposed to MJ.

Preceding experiments on grapes have demonstrated that application of MJ could increase some individual flavonols; however, total flavonol was not influenced (Portu et al., 2016; Ruiz‐Garcia et al., 2012). Flores and Ruiz Del Castillo (2016), by contrast, indicated that preharvest application of 0.2 µM MJ resulted in an increase in total flavonol, although individual flavonols were not affected during storage.

It has previously been reported that spray application of MJ on raspberries and blackcurrants caused quercetin accumulation (Flores & del Castillo, 2015). Since flavonol synthesis follows phenylpropanoid pathway, it is deemed that MJ‐induced PAL activity affects the accumulation of flavonols (Flores & del Castillo, 2014).

Results for resveratrol analysis are illustrated in Table 2. Surprisingly, only a trace of the content of resveratrol was detected in the skin and remained unchanged during the whole storage. This phenomenon was previously explained by Garrido and Borges (2013). These authors stated that resveratrol content is depleted along with maturity. Alleviation of resveratrol content from veraison to maturity contributed to the competition between two biosynthesis pathways triggered by CHS and STS (Jeandet et al., 1995). As can be seen, at day 1 after harvest, resveratrol content enhanced two‐ to threefold by 10 mM MJ treatment. Five and ten millimeter MJ showed an increasing trend during storage (1.7‐ and 3.7‐fold for 5 and 10 mM MJ, respectively). However, the values in 10 mM MJ were much higher than those in 5 mM, indicating that 10 mM was far more effective than other doses. In contrast, resveratrol decreased in 1 mM MJ and disappeared on day 3, suggesting a short‐term effect of a low dose of MJ.

The enhanced transresveratrol level was previously reported for grapes treated with MJ at veraison and 1 week later (Portu et al., 2016). Furthermore, other results from preharvest treatment in different ways, either bunch or foliar application, also confirmed the inducing effect of MJ on stilbene content in both berries and wine (Fernandez‐Marin et al., 2014; Portu et al., 2015). Based on these findings, resveratrol biosynthesis is believed to be stimulated by the MJ‐activated *STS* gene that encodes STS, a pivotal enzyme involved in resveratrol synthesis (Xu et al., 2015). Similar results were also reported through in vitro studies under MJ application (Belhadj et al., 2008; Tassoni et al., 2005). However, further investigation into this research area is warranted.

## 4 CONCLUSION

The present study showed that a single MJ treatment close to the harvest increased phenolic contents during short‐term storage. The effectiveness of the dose depended on the factor evaluated, but 10 mM MJ was more efficient in most factors examined. It seems that this role was accomplished by induction of antioxidant enzymes and the expression of their respective genes, therefore leading to the promoted nutritional values and the decreased oxidative detriment. These findings suggest that the application of MJ in this way could be a useful method for enhancing health‐promoting compounds.

## CONFLICT OF INTEREST

The authors declare that they do not have any conflict of interest.

## ETHICAL STATEMENT

This study does not involve any human or animal testing.

## Data Availability

Data are available within the article.
